# Involvement of *CmWRKY10* in Drought Tolerance of Chrysanthemum through the ABA-Signaling Pathway

**DOI:** 10.3390/ijms17050693

**Published:** 2016-05-11

**Authors:** Muhammad Abuzar Jaffar, Aiping Song, Muhammad Faheem, Sumei Chen, Jiafu Jiang, Chen Liu, Qingqing Fan, Fadi Chen

**Affiliations:** 1 College of Horticulture, Nanjing Agricultural University, Nanjing 210095, China; abuzar_jaffar@yahoo.com (M.A.J.); aiping_song@aliyun.com (A.S.); chensm@njau.edu.cn (S.C.); jiangjiafu@njau.edu.cn (J.J.); 2014204030@njau.edu.cn (C.L.); 2013104100@njau.edu.cn (Q.F.); 2 The State Key Laboratory of Crop Genetics and Germplasm Enhancement, Cytogenetics Institute, Nanjing Agricultural University, Nanjing 210095, China; mlofaheem@gmail.com

**Keywords:** chrysanthemum, drought, abscisic acid, WRKY

## Abstract

Drought is one of the important abiotic factors that adversely affects plant growth and production. The WRKY transcription factor plays a pivotal role in plant growth and development, as well as in the elevation of many abiotic stresses. Among three major groups of the WRKY family, the group IIe WRKY has been the least studied in floral crops. Here, we report functional aspects of group IIe WRKY member, *i.e.*, *CmWRKY10* in chrysanthemum involved in drought tolerance. The transactivation assay showed that *CmWRKY10* had transcriptional activity in yeast cells and subcellular localization demonstrated that it was localized in nucleus. Our previous study showed that *CmWRKY10* could be induced by drought in chrysanthemum. Moreover, the overexpression of *CmWRKY10* in transgenic chrysanthemum plants improved tolerance to drought stress compared to wild-type (WT). High expression of *DREB1A*, *DREB2A*, *CuZnSOD*, *NCED3A*, and *NCED3B* transcripts in overexpressed plants provided strong evidence that drought tolerance mechanism was associated with abscisic acid (ABA) pathway. In addition, lower accumulation of reactive oxygen species (ROS) and higher enzymatic activity of peroxidase, superoxide dismutase and catalase in *CmWRKY10* overexpressed lines than that of WT demonstrates its role in drought tolerance. Together, these findings reveal that *CmWRKY10* works as a positive regulator in drought stress by regulating stress-related genes.

## 1. Introduction

Drought is the most prominent among the abiotic factors that decrease crop production worldwide. Reduced water availability is a limiting factor during the early stage of plant growth and development which reduces the photosynthesis rate; therefore, it has received significant attention from scientists [1,2]. Plants resist these stresses by stimulating stress-responsive genes and regulating physiological and molecular networks and signaling pathways against abiotic stresses [3,4,5]. During unfavorable environmental conditions, abscisic acid (ABA) plays an important role by altering stress-responsive genes [6]. In plants, water-deficit conditions usually result in a high accumulation of ABA that leads to the production of H_2_O_2_ which serves as a signaling intermediate to promote stomatal closure [7,8]. Some of the well-known transcription factor families that play a critical role in abiotic stress especially under drought conditions in many plants include *DREB*, *ERF*, *WRKY*, *MYC*, *MYB*, *bZIP*, *bHLH* and *NAC* [9,10]. Each transcription factor family may trigger a set of genes or provoke a key gene to produce the requisite response needed by the plant.

As one of the largest families of transcription factors, the WRKY protein has a significant role in protecting against stresses such as drought, fluctuating temperatures and salinity. The typical features of WRKY transcription factors include the presence of one or two conserved WRKYGQK motifs at the N-terminal and the zinc finger motif at the C-terminal [4,11,12]. The WRKY protein is involved in the regulation of evolutionary stages of plants such as dormancy, trichome development, seed formation, leaf death, embryogenesis, plant growth and metabolic pathways [5,13,14,15]. Different functions of WRKY have been identified and exclusive binding of this factor to the W-box of target gene promoters is well established and understood in many plant species [15]. Based on characteristic features of the WRKY motif, the entire family can be grouped into three clades (I, II, and III). On the basis of some unique functional motifs in addition to conserved ones, the group II has been categorized into subgroups referred to as IIa to IIe [11]. Many studies in *Arabidopsis* revealed the involvement of the WRKY family in drought-tolerance response. For example, *AtWRKY63* (*ABO3*) gene used ABA-signaling pathway to repress the negative effects of drought [8]. Overexpression of *AtWRKY57* improves drought tolerance by enhancing the ABA level in *Arabidopsis* [16]. Similarly, *AtWRKY25* works in thermo-tolerance by manipulating the transcription of heat shock proteins while *AtWRKY33* confers salt-stress tolerance [17,18]. In addition to this, low Pi level in *Arabidopsis* enhance the activity of *AtWRKY6* to regulate *PHOSPHATE1* expression [19]. *AtWRKY18*, *AtWRKY*40, and *AtWRKY*60 react to ABA and abiotic stress and *AtWRKY70* and *AtWRKY*54 lessen osmotic stress by modifying the stomatal aperture [20,21]. Upregulation of *OsWRKY*30 works against drought tolerance by stimulating MAP kinases [22]. Overexpression of *OsWRKY11* increases tolerance against heat and drought stress under the control of stress-inducible promoter *HSP101*. Similarly, upregulation of *OsWRKY*45 improves the resistance against salt and drought stresses [5,23,24]. Overexpression of *TaWRKY2* improves the tolerance against drought and salt stresses; however, *TaWRKY19* not only alleviates the drought and salt stress but also improves the plant’s tolerance to low temperatures [22,25]. In addition, *TaWRKY10* encourages tolerance to different abiotic stresses by modifying the osmotic balance [26]. Overexpression of the *HvWRKY38* protects against drought and cold stress [27]. Upregulation of *BdWRKY*36 improves the tolerance against drought stress in transgenic tobacco plants [4]. The function of *WRKY* transcription factors has come to be understood due to many studies on the subject; nevertheless, it still requires more research into its role in the floral plant defense mechanism against stresses.

Chrysanthemum (*Chrysanthemum morifolium*) is an important cut flower and pot plant throughout the world. The main problems faced by the chrysanthemum grower are the different ranges of biotic and abiotic stresses [28,29]. Regarding abiotic stresses, one major issue that hampers the production and quality of the cut flowers of chrysanthemums is salinity and drought. Salinity, which is highly correlated with drought in a complex network, makes water unavailable to the chrysanthemum plants, resulting in a low uptake of water by plants from the soil. Subsequently, these conditions produce stunted plant growth, a low number of flower buds, a small flower size, a low number of fully bloomed flowers and flower sticks with a short shelf life [30].

In a previous study, we identified many genes in the WRKY family that play a positive role in regulating the response to drought and salinity stresses [31]. The present study has focused on critically analyzing one gene, namely CmWRKY10 from the WRKY group IIe in response to drought conditions and to determine the signaling pathway associated with this gene.

## 2. Results

### 2.1. CmWRKY10 Belongs to WRKY Group IIe Family

**Figure 1:**
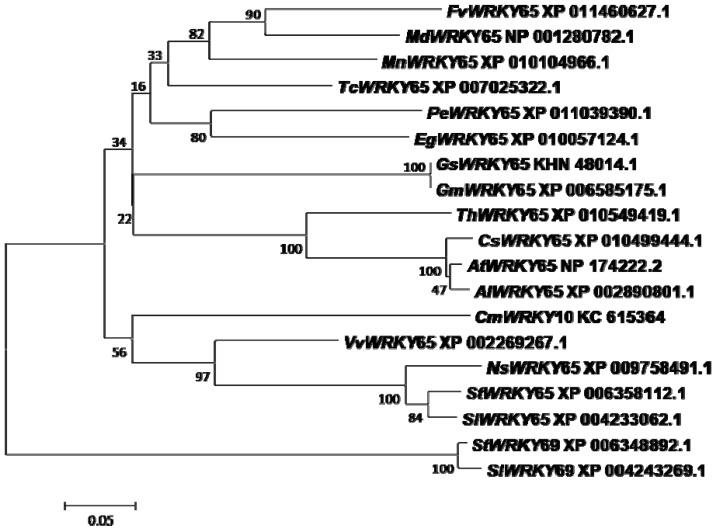
A phylogenetic tree showing the relationship between CmWRKY10 and another plant’s WRKY proteins. At first, amino acid sequences were aligned with ClustalX software after that aligned file was used to construct the phylogenetic tree using neighbor-joining method with 1000 bootstraps in MEGA5 software.

In previous studies, we identified a set of different genes from the WRKY family in chrysanthemum using the RACE PCR technique. Among these genes, *CmWRKY10* was found to be an ortholog of *AtWRKY65*, the open reading frame of which had a full length of 861 bp and encoded 287 amino acids. This amino acid sequence was used to search its ortholog sequence in the NCBI protein database using the BLASTp tool. The analysis yielded 18 ortholog sequences, which were further used for amino acid sequence alignment and phylogeny analysis as shown in Figure 1. Based on the sequences aligned with CmWRKY10, it was determined that CmWRKY10 belongs to the WRKY IIe group family and has characteristics of the transcription factor of AtWRKY65. High conservation with respect to the amino acid, the zinc-finger motif and the presence of the WRKY domain was deduced from the aligned sequence. CmWRKY10 was found to be very close to VvWRKY65, with a similarity of 56%. However, the identity with other orthologs, such as SlWRKY65, StWRKY65, NtWRKY65 and NsWRKY65 was lower than that with VvWRKY65, suggesting that CmWRKY10 has a wide variation and is quite different from the other members of the group.

### 2.2. CmWRKY10 Possess Transcriptional Activity in Yeast Cells

**Figure 2:**
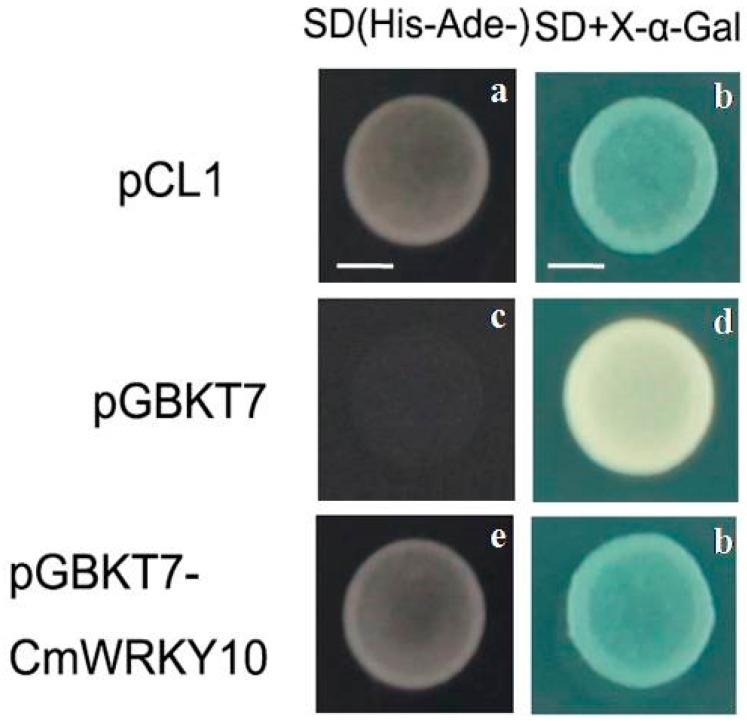
An assessment of the transcription activation assay of CmWRKY10 using the yeast strain and a yeast one-hybrid system. The first column (**a**,**c**,**e**) shows the growth of transformants on SD/-His-Ade selection medium, where the growth of pCL1 (**e**) and empty vector pGBKT7 (**c**) represents the positive and negative control, respectively, representing the transcriptional activity of CmWRKY10; The second column (**b**,**d**,**f**) represents the growth of the same set of colonies in the presence of X-α-gal. Scale bars = 4 mm.

To determine whether *CmWRKY10* possesses transcriptional activity, it was assayed by means of yeast one-hybrid system. As shown in Figure 2, the negative control pGBKT7 was not able to develop on SD/-His-Ade medium, unlike pGBKT7-*CmWRKY10* and the positive control pCL1, which raised well on SD/-His-Ade medium and turned blue on SD+X-α-Gal medium, indicating that *CmWRKY10* has transcriptional activity in yeast cell.

### 2.3. CmWRKY10 Localized in the Nucleus

**Figure 3:**
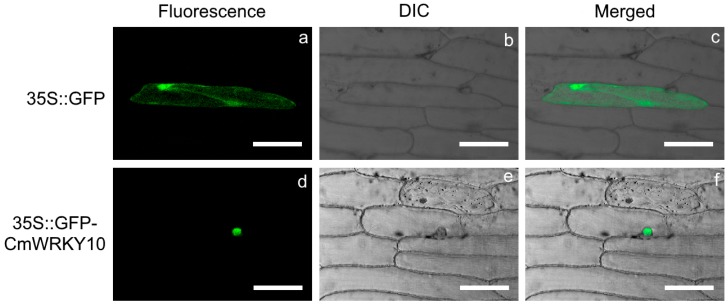
Subcellular localization of CmWRKY10 protein in onion epidermal cell. GFP (upper panel) and GFP::CmWRKY10 (lower panel) fusion proteins were transiently expressed under the control of *CaMV35S* promoter and photographed with Zeizs Microsystem LSM730. Images were taken in dark field for green fluorescence (**a**,**d**), in differential interference contrast (DIC) (**b**,**e**) while the combination of green fluorescence and bright field (**c**,**f**) were also photographed. Scale bars = 50 μm.

Particle bombardment-mediated transient expression assay was used to discover the subcellular localization of CmWRKY10 in onion epidermal cells with the construct *p35S::GFP-CmWRKY10* and a positive vector *p35S::GFP*. The GFP-*CmWRKY10* fusion protein was localized in the nuclei of the onion epidermal cells (Figure 3). In contrast, the control GFP protein lacking CmWRKY10 was dispersed throughout the entire cell. These histological observations proved the subcellular localization of CmWRKY10 in the nucleus to function as a transcription factor.

### 2.4. CmWRKY10 Overexpression Increased Tolerance against Drought in Chrysanthemums

**Figure 4:**
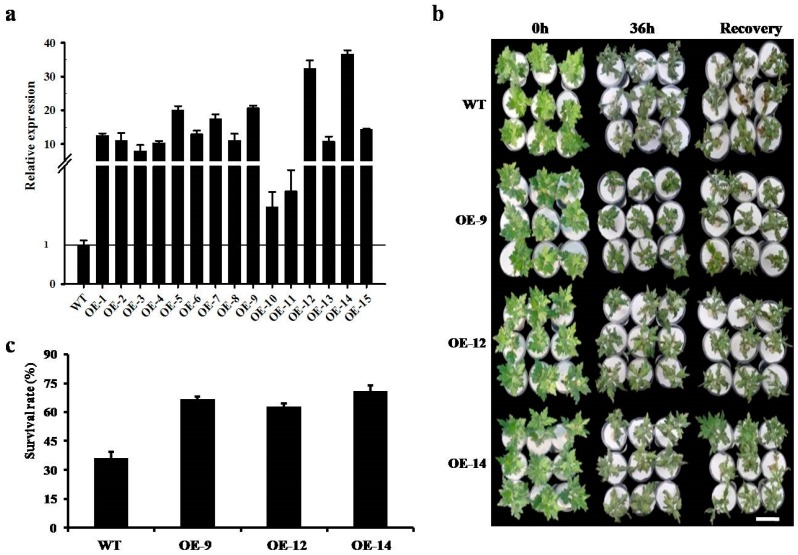
The relative expression level of *CmWRKY10* gene in the overexpressed lines. Results are shown as means ± standard deviation (bars) of three biological repeats; (**a**) phenotypic comparison of*CmWRKY10* overexpressed lines(OE-9, OE-12 and OE-14) and wild-type ‘Jinba’ before and after the drought-stress treatment Scale bars = 60 mm (**b**). The survival rate of the wild-type plants and the overexpressed lines (OE-9, OE-12 and OE-14) after 5 days from a sample size of 65 plants of each individual genotype. Bars represent the standard deviation among three independent replicates (**c**).

The chrysanthemum transgenic lines overexpressing *CmWRKY10* were produced by *Agrobacterium*-mediated transformation. A total of 15 plants were able to regenerate on selection media which were further tested by PCR analysis for positive transformation. The relative expression levels of *CmWRKY10* in all these transgenic lines were assessed using qRT-PCR. The expression level was notably higher in the overexpressed (OE) plants, ranging from 1.96- to 36-fold greater than those in the wild-type (WT) plants. The *CmWRKY10* transcription levels in the lines OE-9, OE-12 and OE-14 were significantly higher than the rest of the OE lines, with expression levels of 20.74-, 32.32- and 36.71-fold greater than WT plants (Figure 4a). Hence, these three lines were selected for further drought-tolerance assessment. The plants from the selected lines were treated with 30% PEG6000 for 48 h and the results showed that the leaves of OE-9, OE-12 and OE-14 plants were less affected. Additionally, most plants survived the stress condition with less wilting of the leaves than the WT plants (Figure 4b). Following the recovery period, the survival rate of OE-9, OE-12 and OE-14 plants were 66.67%, 62.86% and 70.95%, respectively, as compared to WT plants which had the survival rate of 36.19% (Figure 4c). Overall, we can hypothesize that Cm*WRKY10* overexpression improves the drought tolerance in chrysanthemum.

### 2.5. CmWRKY10 Confers Drought Tolerance in Chrysanthemums through Abscisic Acid (ABA) Pathway

**Figure 5:**
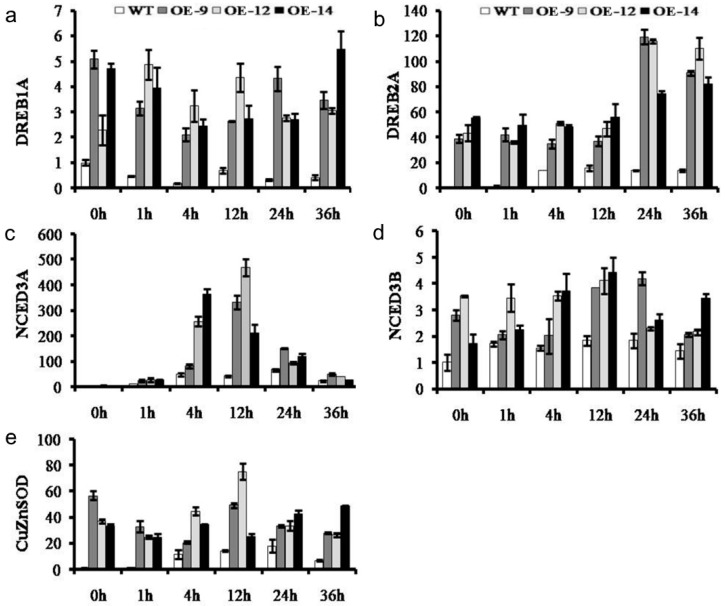
The relative expression of abscisic acid (ABA)-responsive genes *DREB1A* (**a**), *DREB2A* (**b**), *NCED3A* (**c**), *NCED3B* (**d**) and *CuZnSOD* (**e**) in wild-type and transgenic lines at various time points (0, 1, 4, 12, 24 and 36 h). Bars represent the standard deviation among three independent replicates.

To identify the regulatory process of *CmWRKY10* against drought stress, the expression of the ABA-responsive genes *DREB1A*, *DREB2A*, *NCED3A*, *NCED3B* and *CuZnSOD* were assessed. The results showed that *DREB1A* and *DREB2A* were upregulated in the OE lines after 1 h of stress treatment and remained at a high level at all of the time points. In contrast, low expression of *NCED3A* was found in OE lines at 1 h of stress application which sharply increased to reach the maximum expression at 12 h and remained significantly higher than WT plants for the rest of time intervals. In addition, the expressions of *NCED3B* and *CuZnSOD* in WT plants were significantly lower than that in the OE lines following all the treatments (Figure 5). All these results support our hypothesis that the *CmWRKY10* uses ABA-signaling pathway to confer tolerance against drought in chrysanthemum.

### 2.6. Overexpression of CmWRKY10 Reduces Recactive Oxygen Species (ROS) Accumulation due to the Enhanced Activity of Superoxide Dismutase (SOD), Peroxide Dismutase (POD) and Catalase (CAT) under Drought Stress

**Figure 6:**
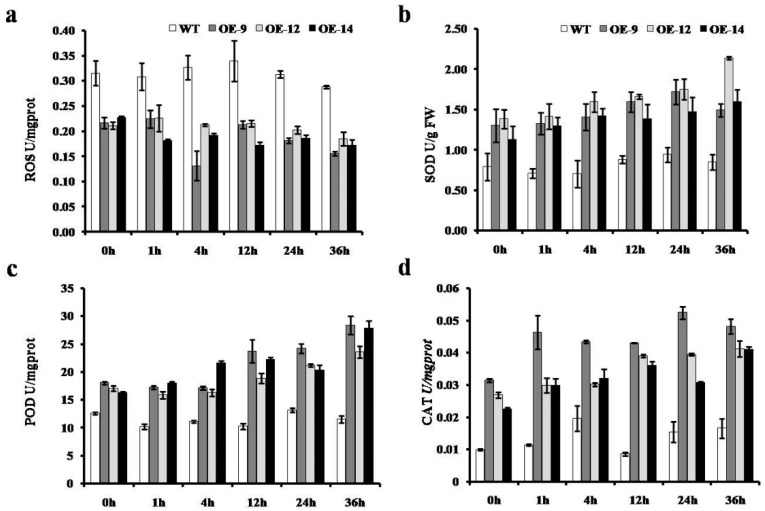
Activities of reactive oxygen species (ROS) (**a**), SOD (**b**), POD (**c**) and CAT (**d**) in the wild-type and overexpressed lines (OE-9, OE-12 and OE-14) under normal and drought conditions at various time points (0, 1, 4, 12, 24 and 36 h). Bars represent the standard deviation among three independent replicates.

To obtain better insight into the function of *CmWRKY10* in chrysanthemum plants under water-deficit conditions, we observed the accumulation of SOD, POD, CAT and ROS in the leaves of *CmWRKY10* OE lines and WT plants at various time points *i.e.*, 0, 1, 4, 12, 24and 36 h. Results shown in Figure 6 reveal that overexpression of *CmWRKY10* lessened the ROS accumulation in OE lines at all of the time points, whereas it increased in WT plants after 1h of stress treatment and remained significantly higher at nearly all of the time points. The results also showed that the activity of three main antioxidant enzymes *i.e.*, SOD, POD and CAT, had increased in OE lines as compared to WT. The value of SOD activity in the WT plants remained at 0.75 U for all of the time points whereas in the OE lines a high accumulation of SOD was observed at all of the time points. In the case of POD, no significant change in the enzyme activity was observed in WT plant during the stress treatment period; however, in all the OE lines, the concentration of POD was significantly higher than WT at all the time points. In OE lines, POD activity continuously increased after treatment and reached its maximum values at 36h post treatment. Results regarding CAT accumulation revealed that in WT plants there were no significant changes observed for CAT concentration during drought treatment as compared to OE lines in which a high concentration of CAT was measured against all time points after drought treatment. Taken together, these results provide strong evidence that *CmWRKY10* decreased ROS accumulation by means of enhanced activity of SOD, POD and CAT to cope with drought conditions.

## 3. Discussion

The chrysanthemum is one of the most important cut flower ornamental plants. Like all other ornamental and crop plants, its growth and productivity are prone to many abiotic and biotic stresses. Among the abiotic factors that critically hamper its growth and deteriorate its quality are salinity and drought. Both of these stresses interact with each other in a complex manner to produce very negative effects on plant growth and the reproduction phase. Therefore, achieving tolerance to these stresses remains a top priority for breeders [32]. Accordingly, this study reported the involvement of one *WRKY* family gene *CmWRKY10* in drought tolerance in chrysanthemum. Our results showed that *CmWRKY10* relates to group II of the WRKY family and is a homolog to *WRKY65* in *Arabidopsis*. The phylogenetic examination of the WRKY proteins revealed that the WRKY family is divided into three groups (I, II and III) with group II being further separated into five subgroups (a to e) [4,33]. Differential expression of the *WRKY* transcription factor critically governs plant developmental and other physiological activities [25]. A large number of *WRKY* genes and their functions have been well known since the identification of the first *WRKY* gene, “*SPF1*”, in sweet potatoes (*Ipomoea batatas*) [34].

The subcellular localization of GFP-*CmWRKY10* depicted that *CmWRKY10-*tagged GFP signals were concentrated only in the nucleus which proved the basis of its functionality as it has been demonstrated in many studies that the transcription factor must reside in the nucleus to perform its activity [4,26,35]. Transcriptional activation analysis illustrated that *CmWRKY10* has transcriptional activity in yeast cells, as reported by Sun *et al.* [4]. The high expression of *CmWRKY10* authenticates the involvement of this transcription factor in alleviating drought stress in chrysanthemum. The analysis of *CmWRKY10* overexpressed lines showed that there is a positive correlation between the expression of *CmWRKY10* and survival rate of the plants when subjected to drought stress (Figure 4b). These findings further corroborate our hypothesis and is in accordance with previous studies. For example, overexpression of wheat *TaWKRY2* and *TaWRKY19* in *Arabidopsis* improved drought tolerance through regulation of the downstream genes [36].

In the literature, there is convincing evidence of ABA’s role in abiotic stresses especially for drought stress. It accumulates in plants under osmotic stress and drought conditions [37]. Different transcription profiles which are involved in regulating abiotic stresses such as less water availability and low temperature in plants are controlled by ABA [10]. Our results revealed that *CmWRKY10* confers drought tolerance in chrysanthemum through the ABA-signaling pathway. The higher expression of the ABA-related gene in the OE plants as compared to WT plants validated this hypothesis. These results are in accordance with previous findings that *WRKY63/ABO3* regulates ABA contents in *Arabidopsis* for drought tolerance in the same way as rice, whereby *OsWRKY45* interacts with ABA-signaling pathway genes to cope with the stress [38]. In addition, this same mechanism was observed for soybean *WRKY20* when expressed in *Arabidopsis*. [38]. Moreover, it is well understood that *DREB* genes, especially *DREB2,* control the drought and salinity-signaling pathway [39]. These genes subsequently regulate key physiological functions involved in the tolerance mechanism [10]. *NCED* is another important enzyme in the synthesis of ABA and it also regulates the drought-stress response [40]. In contrast with the above results, our study showed that the overexpression of *CmWRKY10* improves the survival of chrysanthemum plants more than WT plants by upregulating the ABA-biosynthesis genes *DREB1A*, *DREB2A*, *NCED3A*, *NCED3B* and *CuZnSOD*.

**Figure 7:**
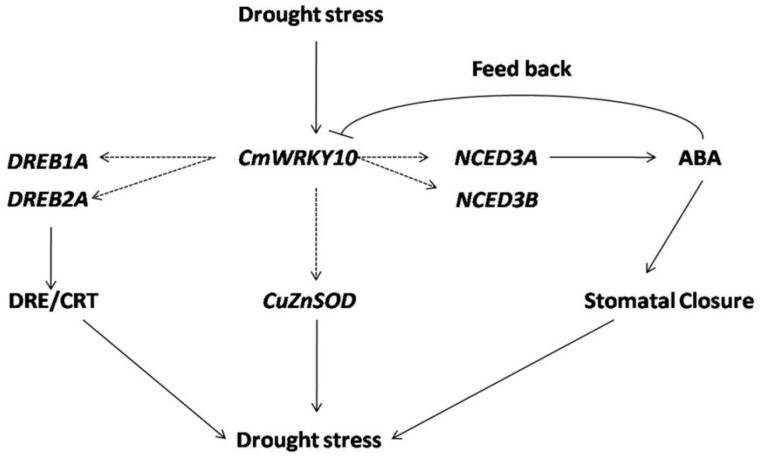
A model of the interaction between *CmWRKY10* and ABA-responsive genes under drought conditions. Solid arrows define the proved interactions while dashed arrows present the proposed interactions based on the results of present study.

Moreover, the accumulation of ABA enhanced ROS activity causing many physiological and metabolic changes in plants to allow them to cope with the stress [37,41]. It has previously been proven in transgenic tobacco plants that wheat TaWRKY10 improved the level of proline and soluble sugar contents in cells by reducing the ROS accumulation to confer tolerance against drought stress [26]. Plants have developed intricate antioxidants such as SOD, POD and CAT to keep them safe from oxidative loss, produced by high accumulation of ROS [42]. In plants, ROS homeostasis is furnished by a complex system of antioxidants which not only scavenge ROS but also protect cells from oxidative damage [43,44]. Some recent studies have shed light on the activity of these antioxidants in relation to WRKY transcription factors for abiotic stresses, especially for drought and salinity. For instance, overexpressing of *DgWRKY3* gene in tobacco resulted in enhanced activity of SOD, POD and CAT to minimize the salt-stress effect [35]. A similar function is executed by the *SlWRKY* gene in tobacco which is effective in both the stresses *i.e.*, drought and salt [16]. In addition, overexpression of the *BdWRKY36* gene in tobacco confers increased tolerance against drought stress due to the increased activity of SOD, POD and CAT [4]. Furthermore, we studied the ROS accumulation in these plants and the outcomes demonstrated that the tolerance of overexpressed lines against drought stress was enhanced because of reduced ROS contents compared with that in WT plants (Figure 6). It is important for the plant to keep the low ROS level to protect itself from oxidative stress and achieves this mostly by triggering the antioxidant activities [45]. Keeping this in view in the present study, the levels of some key antioxidant enzymes like SOD, POD and CAT were measured at different time intervals and the results indicated that the *CmWRKY10* OE plants had enhanced SOD, POD and CAT activity compared to WT plants when subjected to water-stress conditions (Figure 6). These results helped us to speculate that it is the *CmWRKY10* that scavenges the ROS so that OE plants may work more efficiently than WT plants. Collectively, all these results demonstrated that overexpression of the *CmWRKY10* gene may activate the antioxidant defense system resulting in less ROS-mediated injury of transgenic plants under drought stress (Figure 7).

In conclusion, our results show that the *CmWRKY10* from chrysanthemum is a member of group IIe of the WRKY family, which is a positive regulator of drought tolerance. Functional analysis of *CmWRKY10* proved that it causes transcriptional activity by regulating the transcription level of stress-response genes, especially ABA-signaling pathway genes. Future research will be focused on the potential target genes of CmWRKY10 to elucidate the molecular mechanisms associated with *CmWRKY10* mediated stress tolerance.

## 4. Materials and Methods

### 4.1. Plant Material and Growth Conditions

For this experiment, we used the “Jinba” cultivar of chrysanthemum from the Chrysanthemum Germplasm Conservation Center, Nanjing Agricultural University, China. A soil and vermiculite mixture (1:1 *v*/*v*) was used for propagation of identical cuttings of the plant in a greenhouse under standard growth conditions of light and temperature (day/night; 14/10 h, light intensity 50 µmol·m^−2^·s^−1^ and 25/18 °C) with 70% relative humidity.

### 4.2. Sequence Analysis of CmWRKY10

The amino acid sequence from the previous study [31] was used to search for its homolog protein sequences in different dicot species in the NCBI protein database using the BLASTp online tool. All of the sequences with more than 90% coverage were obtained and the sequence alignment of CmWRKY10 was performed along with that of different *WRKY* orthologs of different species using ClustalX software. For the construction of phylogenetic trees neighbor-joining method with 1000 bootstrap was applied, using MEGA5 software.

### 4.3. Transcriptional Activation Analysis in Yeast Cell

The *CmWRKY10* coding sequence without the stop codon was amplified using a Phusion^®^ High Fidelity PCR Kit (New England Biolabs, Ipswich, MA, USA) using the primer pair *CmWRKY10*-GATE-SAL-F/*CmWRKY10*-GATE-NOT-R (Table 1). The amplified product was inserted in the pENTR™1A vector (Invitrogen, Carlsbad, CA, USA) between *Sal* I and *Not* I cloning sites for ligation and recombinant vector was confirmed by sequencing [46]. Both pENTR™1A-*CmWRKY10* and pDEST-GBKT7 were recombined to pDEST-GBKT7-*CmWRKY10* using the LR Clonase™ II enzyme mix (Invitrogen, Carlsbad, CA, USA). The construct pDEST-GBKT7-*CmWRKY10*, pCL1 as positive control, and empty vector pDEST-GBKT7 as negative control were transferred to *Saccharomyces cerevisiae* yeast strain Y2HGold (Clontech, Mountain View, CA, USA) according to the producer’s directions. Collection of the transformants was conducted by either pDEST-GBKT7-*CmWRKY10* or pDEST-GBKT7 using SD/-Trp medium; however, pCL1 was grown in SD/-Leu medium. Yeast cells transformed with pCL1 as the positive control were grown on SD/-His-Ade medium. However, yeast cells with pDEST-GBKT7 could not form a colony on this medium. The clones were then placed on SD/-His-Ade medium supplemented with X-α-gal and incubated at 30 °C for three days before monitoring their ability to spread [30].

### 4.4. Subcellular Localization

Onion epidermal cells were used to detect the subcellular localization of *CmWRKY10*. To construct the GFP::*CmWRKY10*, the complete ORF of *CmWRKY10* was cloned behind the GFP sequence in a pMDC43 vector using the LR Clonase™ II enzyme mixture (Invitrogen). The resulting *p35S::GFP*-*CmWRKY10* construct and empty pMDC43 vector were delivered into onion epidermal cell layers by applying the gold particle bombardment technique [47]. The bombarded onion epidermal layers were incubated under dark conditions for at least 20 h at 24 °C prior to microscopic observation. The GFP signals from onion epidermal layers were detected and visualized by confocal laser microscope.

### 4.5. Chrysanthemum Transformation and Generation of Transgenic Lines

Initially, the complete ORF of *CmWRKY10* was inserted in the plant expression vector pMDC32 to construct the *p35S::CmWRKY10* vector using the LR Clonase™ II enzyme mix (Invitrogen). Then, this *p35S::CmWRKY10* vector was transmuted into EHA105 strain of *Agrobacterium tumefaciens* by applying freeze-thaw transformation technique and following the protocol documented by Li *et al.* [30]. Briefly, young leaves of the chrysanthemum “Jinba” were cut into 0.5 cm diameter pieces and cultured on Murashige and skoog media that contained 8 mg·g^−1^ hygromycine for the selection of positive transformants [48]. The sprouting calli were differentiated into small plantlets following the protocol reported by Cui *et al.* [19]. The successfully regenerated plantlets were then shifted to a greenhouse for further growth under optimal environmental conditions.

The RNA was extracted from OE lines and WT plants after regeneration using RNAiso reagent (TaKaRa, Tokyo, Japan) and RNase-free DNase I (TaKaRa) was used to remove all the traces of DNA. The purified RNA was reverse-transcribed utilizing M-MLV (TaKaRa) enzyme using PCR. For the quantification of *CmWRKY10* expression level, quantitative real-time PCR (qRT-PCR) assay was performed utilizing SYBR^®^ Green reaction kit (TaKaRa) and *CmWRKY10* specific primer pair (CmWRKY10-DL-F/R; Table 1). For the normalization of qRT-PCR data chrysanthemum reference gene *CmEF1α* was used. The outcomes of the transcription (having three biological replicates) were calculated using the 2^–ΔΔ*C*t^ method [49].

### 4.6. Drought Stress Tolerance Assay for Transgenic Lines

A set of 65 cuttings at the 8–10 leaf stage of both transgenic and WT plants was propagated in a 30% PEG6000 solution for 48 h and the roots were then washed and kept in clean water. The plant recovery data were calculated after 5 days [48]. The samples (three biological replications) were collected at specific time points, *i.e.*, 0, 1, 4, 12, 24 and 36 h, after the drought treatment. The samples were kept at −80 °C until further use and RNA isolation.

### 4.7. Expression Profiling of Drought Stress-Related Genes in Overexpressed (OE) Lines of CmWRKY10

To determine the regulatory mechanism of *CmWRKY10* against drought stress, the expression levels of the different stress-responsive genes (*DREB1A*, *DREB2A*, *NCED3A*, *NCED3B* and *CuZnSOD*) were assessed by qRT-PCR. The sequences of all the primer pairs used in this study are listed in Table 1.

### 4.8. Measurements of Physiological–Biochemical Parameters

The ROS content and the activities of POD, CAT and SOD were measured by spectrophotometer utilizing commercial kit (E004, A084-3, A007-1 and A001-4, Jiancheng, Nanjing, China) following the manufacturer’s instructions. Briefly, approximately 0.5 g of chrysanthemum leaves were homogenized in 2 mL of extraction buffer supplemented with 0.1 M phosphate buffer (pH 7.8). Supernatant was separated from samples by centrifuge at 10,000× *g* at 4 °C for 15 min for determination of SOD, POD and CAT enzyme activities.

### 4.9. Statistical Analysis

All of the data recorded for the different physiological parameters were analyzed using one-way analysis of variance (ANOVA) to categorize the difference between the treatments with the least significant difference (LSD) test. For all the statistical analyses the software SPSS v17.0 (SPSS Inc., Chicago, IL, USA) was used.

## Figures and Tables

**Table 1:** The primer names and sequences used in this study.

Primer Name	Sequence (5′ to 3′)
CmHyg-F	CTTCTACACAGCCATCGGTCCAG
CmHyg-R	CGGAAGTGCTTGACATTGGGGAG
Oligo (dT)	AAGCAGTGGTATCAACGCAGAGTACTTTTTTTTTTTTTTTT
dT-R	AAGCAGTGGTATCAACGCAGAGTAC
CmEF1α-F	TTTTGGTATCTGGTCCTGGAG
CmEF1α-R	CCATTCAAGCGACAGACTCA
CmDREB1A-F	CGGTTTTGGCTATGAGGGGT
CmDREB1A-R	TTCTTCTGCCAGCGTCACAT
CmDREB2A-F	GATCGTGGCTGAGAGACTCG
CmDREB2A-R	TACCCCACGTTCTTTGCCTC
CmNCED3A-RT-F	AGTATGGTGGTGAGCCGTTGTATCTAC
CmNCED3B-RT-F	CATACTTGGCGATTGCGGAACCAT
CmNCED3A-RT-R	GCATTCACAATCTGGAGTTCGGACTTC
CmNCED3B-RT-R	GGCTCACCACCATACCTCTCATCAC
CmWRKY10-GATE-SAL-F	CGCGTCGACATGGTGGCTGCATCA
CmWRKY10-GATE-NOT-R	TTTGCGGCCGCGAACATACTTTGA
CmWRKY10-DL-F	TGCTCTTTCGCTCCAACCTG
CmWRKY10-DL-R	TTGTTCAACCAAAACCTCGTCA
CmCuZnSOD-RT-F	CCATTGTTGACAAGCAGATTCCACTCA
CmCuZnSOD-RT-R	ATCATCAGGATCAGCATGGACGACTAC
